# Primary hypothyroidism with exuberant dermatological manifestations^⋆^^⋆⋆^

**DOI:** 10.1016/j.abd.2019.07.010

**Published:** 2020-03-19

**Authors:** Thadeu Santos Silva, Gustavo Baptista de Almeida Faro, Márcia Gabrielle Bonfim Cortes, Vitória Regina Pedreira de Almeida Rego

**Affiliations:** a Dermatology Service, Escola Bahiana de Medicina e Saúde Pública, Salvador, BA, Brazil; b Dermatology Service, Hospital Universitário Professor Edgard Santos, Universidade Federal da Bahia, Salvador, BA, Brazil

**Keywords:** Hypothyroidism, Thyroid diseases, Thyroid gland, Thyroid hormones, Thyroxine

## Abstract

Thyroid hormone has effects on the skin. Patients with hypothyroidism have changes such as dry, scaly and rough skin. Increase carotene in the dermis becomes a yellowish tone to the skin of the patient with hypothyroidism. There is an increase in capillary cycle (anagen phase) and nail growth and a reduction in eccrine gland secretion. It is a case of primary hypothyroidism with nail manifestations associated with dermatologic disorders and successful treatment with levothyroxine. Receptors for thyroid hormone have already been found in keratinocytes, fibroblasts, hair follicles and sebaceous glands. Genes responsive to thyroid hormones and elements of the hypothalamic–pituitary–thyroid axis were identified on the skin. This report highlights the importance of cutaneous manifestations as markers of thyroid disease.

## Introduction

Thyroid hormone effects on the skin.^1^ Patients with hypothyroidism often have cutaneous manifestations such as dry, scaly and rough skin, which may become yellow due to the accumulation of carotene. Dry and brittle hair, thin hair, nail fragility, madarosis and facial edema are common. Edema on the lower limbs or generalized edema can occur, promoting ulcerations and with impact on healing.^2^, ^3^, ^4^

## Case report

**Figure 1:**
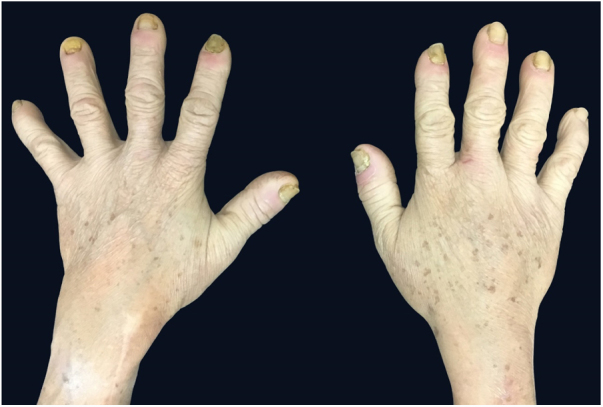
Dystrophic fingernails in quirodactyls.

**Figure 2:**
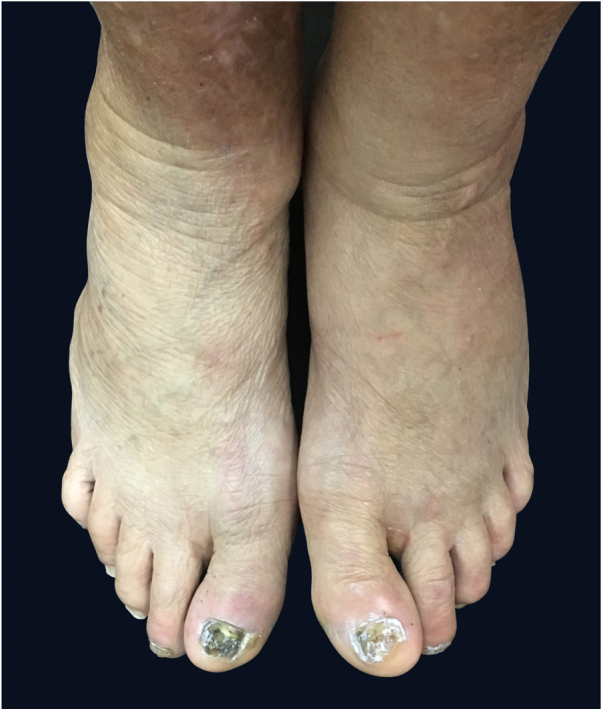
Dystrophic fingernails on toes and malleolar edema.

**Figure 3:**
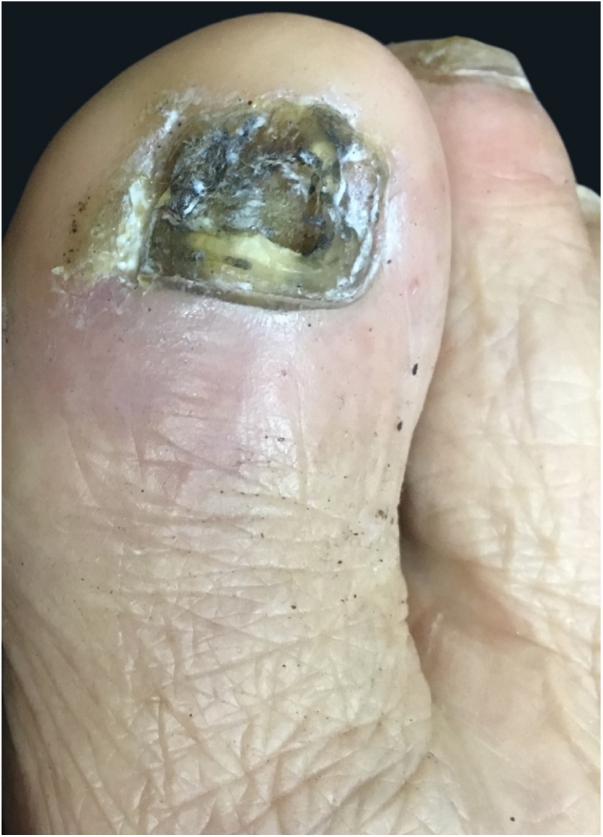
Desquamation and nail pigmentation in the hallux.

**Figure 4:**
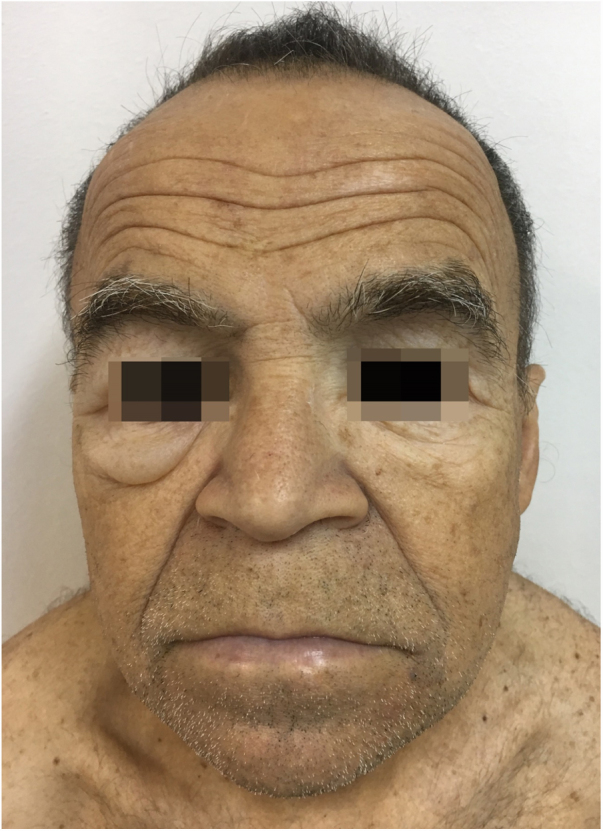
Myxedematous facies, pallor and periorbital edema.

**Figure 5:**
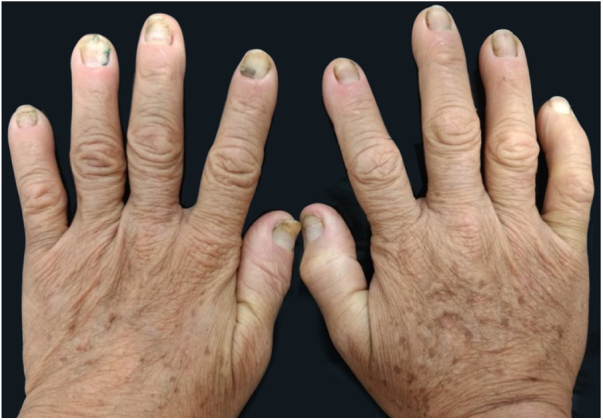
Significant improvement of lesions after 30 days of hormone replacement.

Male, 58-year-old, seller, referring nail dystrophy in all the quirodactyls (Fig. 1) and first on the halux (Figure 2, Figure 3), bilaterally, two years ago. He also presented pallor on mucous membranes, periorbital edema (Fig. 4), more exuberant on the right eye, skin of yellowish and fragile tonality, xeroderma, fissures in palms and plants, asthenia and dizziness with reports of falls. Laboratory investigation showed Hb = 10,7 g/dL, TSH > 50 μU/mL, T4L = 0.65 ng/dL (0.54–1.48 ng/dL), T3 < 0.25 ng/dL, the patient was diagnosed as primary hypothyroidism and received the prescription of levothyroxine. A significant improvement of the symptoms and of the skin was achieved after the first month of treatment (Fig. 5).

## Discussion

Cutaneous manifestations are important as external markers of thyroid disease. Thyroid hormone receptors have been found in keratinocytes, fibroblasts, hair follicles and sebaceous glands. Genes responsive to thyroid hormones and elements of the hypothalamic–pituitary–thyroid axis were identified on the skin. Hypothyroidism reduces the activity of enzymes in the cholesterol sulfate cycle, causes changes in the skin barrier, affects the development of lamellar granules (Odland bodies),^3^ and promotes the accumulation of mucopolysaccharides and water in the dermis. The increased amount of carotene in the dermis induce a yellowish tone to the skin of the patient with hypothyroidism.^3^, ^5^ There is also an increased capillary cycle time (anagen phase) and nail growth and a reduction in eccrine gland secretion.^2^, ^3^, ^4^ Hypothyroidism must be considered in the differential diagnosis of thickened and brittle nails and hair loss, although this extreme presentation is atypical.^4^ For the patient here reported, treatment with levothyroxine sodium was initiated, with significant improvement of symptoms and skin changes already observed at the first month. The presented results show the importance of the dermatologist in the diagnosis of systemic diseases.

## Financial support

None declared.

## Authors’ contributions

Thadeu Santos Silva: Conception and planning of the study; elaboration and writing of the manuscript; obtaining, analysis, and interpretation of the data; critical review of the manuscript.

Gustavo Baptista de Almeida Faro: Elaboration and writing of the manuscript; intellectual participation in the propaedeutic and/or therapeutic conduct of the studied cases; critical review of the literature.

Márcia Gabrielle Bonfim Cortes: and planning of the study; obtaining, analysis, and interpretation of the data; intellectual participation in the propaedeutic and/or therapeutic conduct of the studied cases; critical review of the manuscript.

Vitória Regina Pedreira de Almeida Rego: of the final version of the manuscript; elaboration and writing of the manuscript; effective participation in research orientation; critical review of the literature.

## Conflicts of interest

None declared.
